# 

**Published:** 1920-07-17

**Authors:** 


As a result of a Garden Fête arranged by the Highgate Division of the League of the Roses, the sum of £625 has been handed to the Great Northern Central Hospital. By permission of Mr. and Mrs. Albert Barratt (who defrayed the entire expenses) the function was held at their residence, Totteridge Park, Herts.

### Hospital Sunday, 1920.

The total of the Hospital Sunday Fund celebrations to July 7 was £60,000. Included in this are donations from £4,000 to £20, and church collections as follows: St. Columbas (Church of Scotland), Pont Street, £670; St. James's, Piccadilly, £394; Holy Trinity, Sloane Street, £370; Christ Church, Lancaster Gate, £335; St. Michael's, Cornhill, £314; St. Alphage, London Wall, with St. Mary, Aldermanbury, £206; Westminster Chapel, Buckingham Gate, £206; St. Jude's, South Kensington, £175; Brixton Independent Church, £174; St. Ann's, Stamford Hill, £150; Whitechapel Primitive Methodist Mission, £150; St. Andrew's, Lambeth, £147; Great Stanmore Parish Church, £145; St. Mary's, Islington, £139; St. Anne's, Soho, £134; St. Jude's, Mildmay Park, £128; Immaculate Conception, Farm Street, £127; All Saints', Margaret Street, £125; St. Philip's, Kensington, £116; St. Peter's, Belsize Park, £116; Christ Church, Gipsy Hill, £104; St. Luke's, Redcliffe Square, £104; St. Bartholomew's, Sydenham, £103; Temple Church, £102; Christ Church, Westminster, £101.

The directors of the London County Westminster and Parr's Bank, Ltd., have declared an interim dividend of 10 per cent. for the half-year ended June 30 on the £20 shares, and the maximum dividend of 6¼ per cent. on the £1 shares for the same period.

### Matrons' and Sisters' Appointments.

Booth Hall Infirmary for Children, Blackley, Manchester.—Miss Annie Shaw has been appointed night superintendent. She was trained at Oldham Union Infirmary, and has since been ward sister at Booth Hall Infirmary. Miss Shaw has also done private and district nursing.

City Hospital, Grafton Street, Liverpool.—Miss Laura Yaxley has been appointed sister. She received her fever training at the above-named institution, and her general training at the Mill Road Infirmary, Liverpool. Miss Yaxley has been staff nurse at Bethnal Green Infirmary, and has also done Army nursing.

Isolation Hospital, Boscombe, Bournemouth.—Miss Mary Winstanley has been appointed sister. She was trained at Nottingham General Hospital, has held an appointment at Brighouse Fever Hospital, has served under the British Red Cross Society, and has done private nursing.

Phthisical Sanatorium, Maiden Law, near Lanchester.—Miss Kathleen Madge Nelsen has been appointed matron. She was trained at the Royal Infirmary, Newcastle, and at the City Hospital, Birmingham. She is at present health visitor in Newcastle.

Reigate and Redhill Hospital, Redhill.—Miss Agnes Fortescue Reade has been appointed matron. She was trained at St. Thomas's Hospital, where she was later sister, assistant housekeeper, and night superintendent; has been sister-matron of a private nursing home at Woking, and night superintendent at the Seamen's Hospital, Greenwich.

Scottish Homœopathic Hospital for Children, Mount Vernon, near Glasgow.—Miss Lily A. Gostling has been appointed matron. She was trained at the London Hospital, where she has since been night sister and sister-in-charge of the women's medical ward, and has also been assistant matron of the Babies' Castle, Hawkhurst.

### Passing Events and Topics.

### Child Welfare at Walthamstow.

The report of the Walthamstow Child Welfare Society, presented at the recently held Annual Meeting, shows a steady increase of useful work in this densely populated district. At the Centre, Branch, and Weighing Station there had been an increase in all branches of the work. Lectures had also been given to mothers at the Centre, and the needlework classes had been well attended. Following upon this successful year the Society is organising a "Baby Week," opening on July 1 with a Garden Party and Sale of Work; on July 2, Dame Mary Scharlieb will deliver a lecture in the County High School; Saturday will be a "Flag Day" in aid of the local funds; and on Sunday, July 4, special sermons will be preached in the local churches, the "Week" being brought to a close on Monday by a "Mother's Day" at "Brookscroft," when those attending the Centre will be suitably entertained.

